# A Composite Anion Conducting Membrane Based on Quaternized Cellulose and Poly(Phenylene Oxide) for Alkaline Fuel Cell Applications

**DOI:** 10.3390/polym12112676

**Published:** 2020-11-12

**Authors:** Dong Ho Kang, Gautam Das, Hyon Hee Yoon, Il Tae Kim

**Affiliations:** 1 Department of Chemical and Biological Engineering, Gachon University, Seongnam-si 461-701, Korea; kdh459@naver.com; 2 Department of Chemical Engineering, Hanyang University (Erica Campus), Ansan-si 15588, Korea; gautam2706@gmail.com

**Keywords:** anion exchange membrane, urea fuel cell, quaternized cellulose, composite, cross-linking

## Abstract

In this study, composite anion exchange membranes (AEMs) were synthesized by cross-linking poly(phenylene oxide) (PPO) with cellulose functionalized by 1,4-diazabicyclo[2.2.2]-octane (DABCO) or di-guanidine (DG). The structural and morphological characteristics of the synthesized AEMs were characterized by FTIR, ^1^H-NMR, SEM, TEM, and AFM, while their performance was evaluated in terms of ionic conductivity, water uptake, ion exchange capacity, and tensile strength with respect to the loading of the quaternized cellulose in the quaternized PPO (qPPO) matrix. The composite AEMs exhibited considerably enhanced mechanical and alkaline stability as well as good anion conductivity. The composite AEM with 7 wt% of cellulose functionalized with DG in the qPPO matrix (qPPO/DG-Cel7) exhibited a maximum hydroxide conductivity of 0.164 S cm^−1^. Furthermore, a urea/O_2_ fuel cell prepared using this composite membrane showed a maximum power density of 12.3 mW cm^−2^. The results indicated that the cellulose-based composite membranes showed a satisfactory performance in alkaline fuel cell applications.

## 1. Introduction

Anion exchange membrane fuel cells (AEMFCs), as low-temperature fuel cells, have garnered lots of research interest over the past decade because of their endowed advantages such as low-cost catalyst system, fast oxygen reduction kinetics in alkaline solutions, and generation of environmentally benign by-products [1,2,3,4]. In AEMFCs, the anion exchange membrane (AEM) is a key component which acts as an electronic insulator and ionic conductor at the same time. However, the major drawbacks of AEMs are its low ionic conductivity and alkaline stability at elevated temperatures [5,6,7]. Since high ionic conductivity is required to obtain high power output, low ionic conductivity due to low hydroxyl ion mobility and high pK_b_ of a quaternary amine of an anion exchange membrane must be addressed [2,8]. Primarily, the ionic conductivity of the AEM is directly related to the fixed cationic groups and the hydration level [9]. To achieve high ionic conductivity, strategy such as increasing the ion exchange capacity (IEC) by multication functionalization of the polymer [10] or inducing the phase-segregated morphology by graft polymerization [11], block-copolymerization [12,13], comb-shaped copolymers [14], and composite membranes with different nano/micro fillers [15,16] were investigated. Typically, composites which consist of polymer matrix and immiscible filler have gained advantage of obtaining a good electrochemical performance and controlling the ionic conductivity by simply changing the composition of the filler [17,18]. However, it is to be noted that high IEC will inevitably result in high water uptake which detrimentally affects the membrane stability [19]. Consequently, cross-linking might enhance the mechanical and alkaline stability while maintaining high ionic conductivity [20,21,22]. Li et al. reported enhanced ionic conductivity by cross-linking between the multiblock ionomer and nano-filler [23]. Thus, cross-linking the fillers in the polymer matrix will avert excessive swelling of AEMs and prevent leaching or agglomeration of the fillers in the matrix thereby maintaining fine stability [24].

Recently, polyphenylene oxide (PPO) has attracted considerable interest as AEM material in AEMFCs owing to its excellent thermal property, good mechanical attributes, and chemical stability [25,26]. PPO has been widely applied in various fields such as high-pressure hydraulic fracturing [27,28], porous media [29,30], and pervaporation membrane [31,32].

Cellulose has been widely used in different applications such as batteries, metal removal, and biomedical fields, however it has not been extensively investigated for AEM applications [33,34,35]. Cellulose possesses large surface area, high tensile properties, good chemical stability, and aptitude for facile and selective modification at the hydroxymethyl group in the C-5 position [18,36,37,38,39]. Cellulose’s compatibility and dispersibility with polymer matrices were reported to be achieved with the appropriate functionalization of cellulose, which will increase the IEC of the AEMs and also increase the thermal and mechanical properties of the AEMs [39,40,41]. Cheng et al. enhanced the ionic conductivity of AEMs by incorporating quaternized cellulose nanocrystal into the polymer matrix [42]. Thus, functionalization of cellulose and cross-linking with the polymer chains can effectively enhance the interfacial interaction with the polymer matrix [43]. Furthermore, different kinds of cationic groups were investigated such as imidazolium [44], phosphonium [45], and quaternary ammonium [46,47]. DABCO is known to be stable under alkaline solution with its conformational characteristic [48] and DG is expected to have good ionic conductivity and alkaline stability due to its strong basicity and resonance structure [49].

Hence, the quaternization of cellulose with DABCO and DG could be expected to not only increase the IEC of qPPO-based AEMs but also provide contact points for cross-linking, which eventually improves the ionic conductivity and alkaline stability. In this study, a novel composite membrane has been synthesized, and the compositional ratio of qPPO/quaternized cellulose was optimized with respect to ion exchange capacity, water uptake, swelling ratio, ionic conductivity, alkaline stability, and mechanical properties. The performance of a urea/O_2_ fuel cell comprising the synthesized AEMs was evaluated.

## 2. Materials and Methods

### 2.1. Materials

Poly(2,6-dimethyl-1,4-phenylene oxide) (PPO), N-bromosuccinimide (NBS), azobisisobutyronitrile (AIBN), Sigmacell cellulose (Cel), triethylamine (TEA), p-toluenesulfonyl chloride (PTSC), 1,4-diazabicyclo[2.2.2]octane (DABCO), 1,1,3,3-tetramethylurea, 1,4-dibromobutane (DBB), chlorobenzene, *N*-methyl-2-pyrrolidone (NMP), *N*,*N*-dimethylformamide (DMF), dichloromethane, *N*,*N*-dimethylacetamide (DMAc), acetonitrile, ethyl acetate, and toluene were purchased from Sigma-Aldrich, Yongin, Korea. Lithium chloride (LiCl) was purchased from Alfa Aesar, Incheon, Korea. An oxalyl chloride (OC) and 1,6-diaminohexane were purchased from DAEJUNG, Siheung, Korea. All chemicals were of reagent grade.

### 2.2. Synthesis of DG

Guanidine and DG were synthesized as described in a previous report (Supplementary Scheme S1) [49]. To prepare guanidine, 1,1,3,3-tetramethylurea (0.64 g) was first dissolved in dichloromethane (10 mL) at room temperature. The clear solution that was formed was cooled to 0 °C. Then, OC (1 g) was added in aliquots amount under constant stirring and nitrogen purging. The temperature was then slowly raised to 60 °C and maintained for 2 h. The guanidine that was formed as a result was then dissolved in 5 mL of acetonitrile under nitrogen conditions. To this, 1,6-diaminohexane (0.29 g) and TEA (0.56 g) were slowly and sequentially added at 0 °C. The reaction was allowed to continue for 18 h at 90 °C. The reaction mixture was then precipitated in 20 mL of NaOH (5 mol L^−1^) aqueous solution and washed with dichloromethane. Finally, the obtained material was dried overnight in a vacuum oven.

### 2.3. Quaternization of Cellulose

Tosylation of cellulose was carried out in a DMAc/LiCl solvent [50]. Cel (1.2 g) was dissolved in a solvent system containing DMAc (52 mL) and LiCl (3.68 g) at 70 °C for 4 h under an inert atmosphere. The temperature of the above solution was then adjusted to 2–4 °C using a chiller. Then, TEA (9.37 mL) and PTSC (7.822 g) were added to the reaction flask and stirred for 24 h. The above solution was precipitated in ice-cold water and stirred vigorously for 15 min. The precipitate was then washed with methanol and was lyophilized. The obtained dried tosylated cellulose was denoted as t-Cel.

**Scheme 1:**
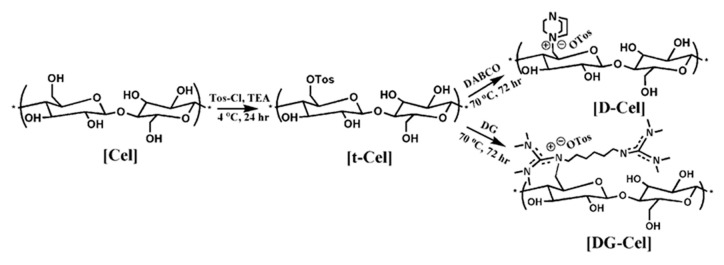
Quaternization of cellulose.

Next, the quaternization of t-Cel was carried out using DABCO or DG in DMF. Firstly, t-Cel (2.06 g) was dissolved in DMF (40 mL) under an inert atmosphere at 70 °C. Then, DABCO or DG was added in aliquots (10 mole equivalents). The quaternization reaction was carried out for 72 h, after which the entire system cooled to room temperature and was precipitated in diethyl ether (Scheme 1). The precipitate was washed with ethyl acetate and diethyl ether several times and was dried overnight in a vacuum oven. The obtained quaternized celluloses with DABCO or DG were denoted as D-Cel and DG-Cel, respectively.

### 2.4. Quaternization of PPO

Firstly, the brominated PPO was prepared using a slightly modified version of a previous reported method [51]; briefly, PPO (6 g) was completely dissolved in chlorobenzene (60 mL) under vigorous stirring, and the temperature was slowly raised to 140 °C. Afterward, AIBN (0.25 g) followed by NBS (4.45 g) were added to the above solution and stirred for 5 h in an inert atmosphere. Then, the reaction mixture was precipitated and washed by pouring into a 10-fold excess of methanol. The dark precipitate was dissolved in chloroform and reprecipitated in methanol. This was again washed repeatedly with methanol and dried overnight in a vacuum oven at 45 °C to obtain brominated PPO (bPPO).

**Scheme 2:**
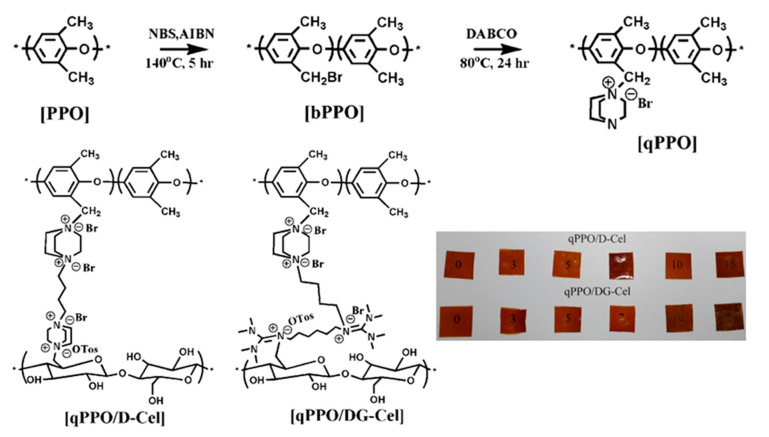
Quaternization of polyphenylene oxide (PPO) and fabrication of composite membrane.

The quaternization of bPPO (2 g) was carried out by dissolving it in NMP (40 mL), and then DABCO (4 mole equivalent) was added to the above solution. The reaction was carried out at 80 °C for 24 h under a nitrogen atmosphere. The reaction mixture was repeatedly precipitated and washed with toluene. Finally, the product was dried overnight in a vacuum oven at 45 °C to obtain quaternized PPO (qPPO) (Scheme 2). The theoretical molecular weight of qPPO was calculated to be 233.17 g mol^−1^.

### 2.5. Fabrication of the Composite Membrane

The composite membranes were prepared using different weight percentages of quaternized cellulose in the range of 0–15 wt%, as shown in Supplementary Table S1. A clear solution of qPPO (1 g) was first prepared in NMP (6 mL), to which a solution of quaternized cellulose dispersed in NMP (2 mL) was added to the desired wt% (Supplementary Table S1). The quaternized cellulose with qPPO solution was vigorously stirred at 80 °C for 24 h. The whole solution was then transferred into a tightly sealed container and 240 µL of DBB was added and stirred for 15 min at 80 °C. The pre-gelled liquid was poured into a glass petri dish to obtain membranes of uniform thickness after the solvent was completely evaporated at 80 °C. These membranes were denoted as qPPO/D-Cel or qPPO/DG-Cel (qPPO/quaternized cellulose) along with a number (0 to 15), which indicated the weight percentage of the quaternized cellulose. Scheme 2 illustrates the formation of the composite membranes.

### 2.6. Characterization

Fourier transform infrared spectrometry (FTIR; JASCO FT-IR 300E) was used to record the FTIR spectra, while nuclear magnetic resonance (NMR) spectroscopy (400 MHz ^1^H-NMR Spectrometer, BrukerBioSpin) was used to analyze the ^1^H-NMR spectra of the samples. The morphological characterization was carried out by scanning electron microscopy (SEM, Hitachi S-4700, Tokyo, Japan), transmission electron microscopy (TEM, TecnaiG2 F30 S-Twin, AP Tech), and atomic force microscopy (AFM, XE-150, Park Systems, Suwon, Korea). The TEM samples were prepared by ultramicrotomy (UMT, PT PC Ultramicrotome and Photographic, RMC, New York City, NY, USA) immersed in Na_2_WO_4_ (1 mol L^−1^) solution at 30 °C for 48 h. The samples were then washed several times with HPLC water and dried in a vacuum oven at 30 °C for 24 h. Thermogravimetric analyzer (TGA, TA Instruments SDT Q600, Philadelphia, PA, USA) was used to investigate the thermal stability of the membranes. The equations used in a previous work [25] were employed to calculate the IEC, water uptake, and swelling ratio of the membranes.

### 2.7. Ion Exchange Capacity (IEC), Water-Uptake (WU), Swelling Ratio (SR), and Gel Fraction (GF)

The IEC was determined by the acid-base titrimetric method. Briefly, the anion exchange membranes were cut into specific dimensions and soaked in HCl (0.01 mol L^−1^) for 24 h. Then, the acid-base titration was carried out with NaOH (0.01 mol L^−1^) using phenolphthalein indicator. The IEC was then calculated by measuring the dry weight of the membrane and using the following equation:

(1) $$\mathrm{IEC}=\;\frac{\left(V_{a}-V_{b}\right)\times C_{NaOH}}{W_{d}}$$

where $V_{a}$ is the volume of the blank sample, $V_{b}$ is the volume of the titrated sample, $C_{NaOH}$ is the concentration of NaOH used for titration, and $W_{d}$ is the dried weight of the membrane.

The WU and SR were measured by comparing the wet and dried states of the membranes at room temperature. A series of membranes were cut, and their weight, width, and thickness were precisely measured in both dry and wet states. Then, the WU, in-plane SR (SR_ip_), and through-plane SR (SR_tp_) were calculated using the following equations:

(2) $$\mathrm{WU}=\left(\frac{W_{w}-W_{d}}{W_{d}}\right)\times 100\;\left(\%\right)$$

(3) $${\mathrm{SR}}_{\mathrm{ip}}=\left(\frac{L_{w}-L_{d}}{L_{d}}\right)\times 100\;\left(\%\right)$$

(4) $${\mathrm{SR}}_{\mathrm{tp}}=\left(\frac{T_{w}-T_{d}}{T_{d}}\right)\times 100\;\left(\%\right)$$

where $W_{w}$ and $W_{d}$ represent the weights, $L_{w}$ and $L_{d}$ represent the widths, and *T_w_* and *T_d_* represent the thickness of wet and dried membranes, respectively.

The GF was evaluated by measuring the weight of the membrane before and after soaking into NMP (20 mL) for three days at 70 °C. All samples were weighed in a fully dried state and the membranes were cut to prepare equal-sized specimens; the GF was calculated according to the following equation [52]:

(5) $$\mathrm{GF}=\;\frac{W_{a}}{W_{b}}\times 100\;\left(\%\right)$$

where $W_{b}$ and $W_{a}$ represent the dried weight of the membrane before and after soaking in NMP, respectively.

### 2.8. Ionic Conductivity (σ)

Frequency response analyzers (Solartron 1255B and 1287) were used to obtain the Nyquist plot of the hydroxide-exchanged membranes by a two-point probe technique. The Nyquist plot was obtained in the frequency range of 100 Hz to 700 kHz. Each membrane sample (2.5 cm × 2.5 cm) was placed in the conductivity cell and tightly screwed for maximum contact with the electrodes in single cell compartments. Humidified nitrogen gas was introduced into the unit cell apparatus to prevent CO_2_ contamination and desertification of the membranes. The ionic conductivity was calculated by the equations below [25]:

(6) $$\sigma =\;\frac{d}{R\times A}$$

where σ is the ionic conductivity (S cm^−1^), *d* is the distance between the two electrodes (cm), *R* is the resistance (ohm), and *A* is the surface area (cm^2^) of the membranes.

### 2.9. Tensile Measurements and Alkaline Stability

The tensile strength of the membranes in the wet state was measured using a low-load universal testing machine (UTM; JSV-H1000, JISC, Tokyo, Japan). The membrane samples were cut into dimensions of 2 cm × 4 cm and were held between the two jaws of the UTM. The tensile strength was then measured at a crosshead speed of 1 mm min^−1^ at room temperature.

To determine their alkaline stability, the AEM samples were soaked in KOH solution (1 mol L^−1^), which was saturated with nitrogen to avoid CO_2_ contamination at 60 °C for 120 h. After soaking, the ionic conductivity and IEC were calculated, and these values were used to determine the alkaline stability.

### 2.10. Electrode Preparation and Fuel Cell Analysis

Commercial Ni/C (20%, Fuel Cell Store) and Pt/Ru (60%, atomic ratio 1:1, Fuel Cell Store) were used as the anode and cathode catalysts, respectively. The catalysts were dispersed in 1-propanol solution in de-ionized water and mixed with an anion ionomer solution (Fumion FAA-3-SOLUT-10, Fuel Cell Store). The prepared catalyst ink was then coated onto a carbon paper (Toray carbon paper 120, wet proofed, Fuel Cell Store) at loading of 5 and 1.5 mg cm^−2^ for the anode and cathode, respectively. The synthesized AEM was put between the electrodes and pressed at room temperature for 1 min to obtain a membrane electrode assembly with an active area of 5.0 cm^2^. A graphitic plate with serpentine flow channels and gold-coated stainless current collectors were used for the single-cell test. An aqueous solution of urea (0.33 mol L^−1^) in KOH (3 mol L^−1^) was fed into the anode side at a rate of 15 mL min^−1^, while humidified oxygen was supplied to the cathode at a rate of 200 mL min^−1^. Fuel cell performance was evaluated utilizing a potentiostat/galvanostat (VSP, Bio-Logic, Seyssinet-Pariset, France) interfaced with EC-lab 11.01 data acquisition software.

## 3. Results and Discussion

### 3.1. Characterization and Morphological Studies

**Figure 1:**
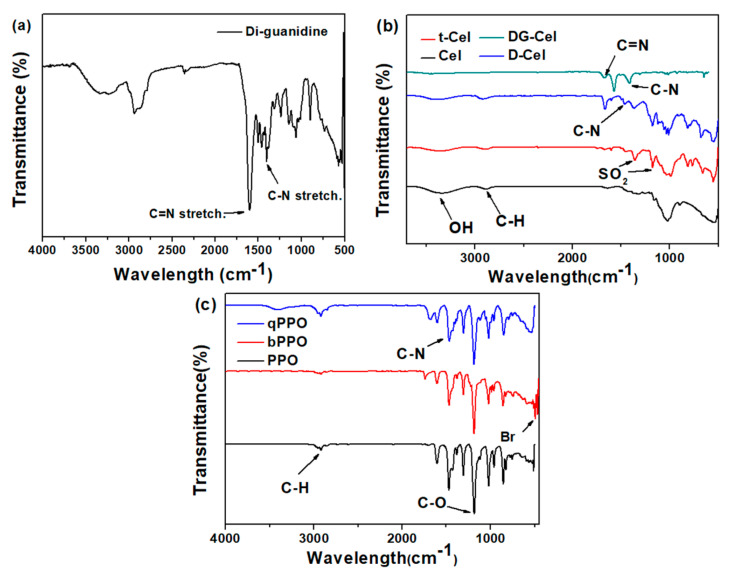
FT-IR spectra of (**a**) di-guanidine (DG), (**b**) sigmacell cellulose (Cel), and (**c**) PPO.

Figure 1 illustrates the FTIR spectra of DG, Cel, and PPO. A strong absorption band at 1610 cm^−1^ corresponding C=N stretching vibration and a band 1400 cm^−1^ for C–N stretching were observed in the FTIR of DG (Figure 1a) as reported previously [53] The FTIR spectra of cellulose, tosylated cellulose, and quaternized cellulose are shown in Figure 1b, revealing that the -OH stretching band of pristine cellulose diminished in intensity after tosylation reaction. Moreover, asymmetric $S=O_{2}$ stretching at 1355 cm^−1^, and symmetric $S=O_{2}$ stretching at 1177 cm^−1^, were observed in the FTIR spectra of tosylated cellulose, which confirmed the tosylation reaction. After the quaternization reaction, the $S=O_{2}$ stretching band diminished and a band located at 1431 cm^−1^ indicated substitution of the tosyl group by DABCO [18]. Similarly, DG-Cel showed the stretching band corresponding to the DG at 1610 cm^−1^ for the C=N stretching, confirming the quaternization reaction [54]. As shown in Figure 1c, the characteristics of the stretching band for PPO were observed at 2923 (aromatic C–H stretching), 1469, and 1600 (aromatic ring stretching), and 1182 cm^−1^ (aromatic C–O stretching) [25]. The bromination of PPO was confirmed by the C-Br stretching band at 500 cm^−1^ in the FTIR spectra of bPPO (Figure 1c) [51]. The C-Br stretching band diminished after quaternization, and, additionally, the C-N stretching band at 1431 cm^−1^ appeared in the FTIR of qPPO, confirming the quaternization of bPPO [51].

The NMR spectrum of DG is presented in Supplementary Figure S1a. The peak of 2.92 ppm shows the chemical shift of proton, which is attached to the nitrogen, and 1.3, 1.7, and 3.6 ppm indicate the methyl proton located at a long alkyl chain in the middle of the DG molecule [49]. The ^1^H-NMR spectra of pristine Cel, t-Cel, D-Cel, and DG-Cel are shown in Supplementary Figure S1b–e. Cellulose shows a broad peak in the range of 3.9–6.0 ppm. It has been reported that hydrogen bonding in cellulose leads to chemical shifts, and peak broadening [55]. Thus, the proton resonance of the glucose ring in cellulose can be shifted to higher ppm corresponding within the broadened peak (3.9–6.0 ppm) [50]. In the case of t-Cel, additional peaks corresponding to the tosyl group were observed at 7.4 ppm and 7.8 ppm, indicating tosylation of cellulose [56]. Furthermore, after quaternization, new peaks at 3.0 ppm and 2.7 ppm confirm the quaternization of D-Cel and DG-Cel, respectively [49,53].

The ^1^H-NMR spectra for PPO, bPPO, and qPPO are presented in Supplementary Figure S1f–h. In the case of PPO, the peak at a chemical shift value of 2.0 ppm was due to the methyl group and at 6.5 ppm was due to the benzene ring protons [26]. A new intense peak was observed at 4.2 ppm after the bromination of PPO, which indicated the existence of the C-Br group in bPPO [57]. Subsequently, the peak at 4.2 ppm disappears after the quaternization reaction and additional peak corresponding DABCO was observed at 3.0 ppm, which indicates the successful attachment of DABCO to the bPPO [49,53].

**Figure 2:**
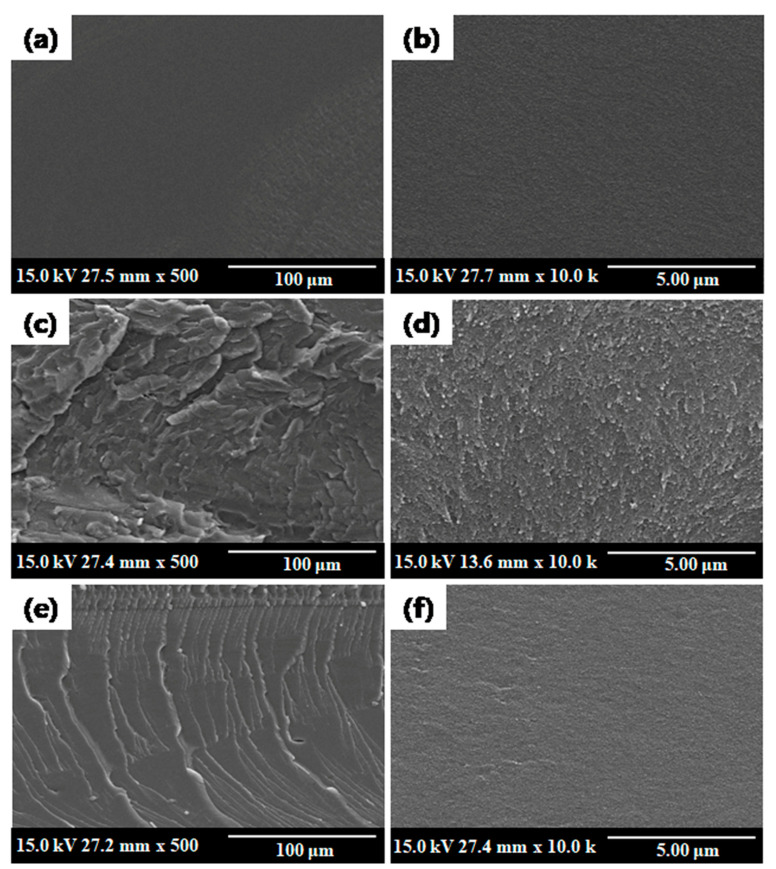
Cross-sectional SEM images of quaternized PPO (qPPO) (**a**,**b**), qPPO/D-Cel7 (**c**,**d**), and qPPO/DG-Cel7 (**e**,**f**) membranes.

The cross-sectional SEM images of qPPO, qPPO/D-Cel7, and qPPO/DG-Cel7 are presented in Figure 2. As shown in Figure 2c–f, when comparing images of qPPO without filler Figure 2a,b, no visible macroscopic phase segregation was observed, indicating the homogeneous dispersion of the quaternized cellulose fillers (i.e., D-Cel and DG-Cel) in the membranes. It was also observed that the morphology of the pristine cellulose remains intact despite the subsequent chemical modification, i.e., it retains the original structure without structural breakdown (Supplementary Figure S2). The length of cellulose was measured by SEM. The Cel’s length range was 4.7 to 18.8 μm, 12.2 to 15.5 μm for t-Cel, and 13.1 and 20.1 μm for D-Cel and DG-Cel, respectively.

**Figure 3:**
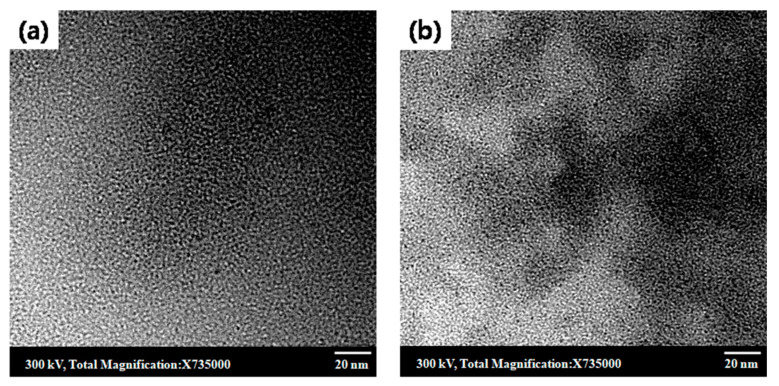
TEM images of (**a**) qPPO/D-Cel7, (**b**) qPPO/DG-Cel7, and both are stained with WO_4_^2−^.

The structure of the membranes was further characterized by TEM as shown in Figure 3. The dark regions in the TEM image correspond to the hydrophilic domain, whereas the bright region represents the hydrophobic part [25,58,59]. The distribution of the darker regions in qPPO/DG-Cel7 (Figure 3b) is more distinct and evenly spread than in qPPO/D-Cel7 (Figure 3a), demonstrating better phase segregation in the former. Furthermore, the AFM images of qPPO/D-Cel7 and qPPO/DG-Cel7 (Supplementary Figure S3) display a distinct morphology. Previous reports indicated that the bright and dark region in the AFM images corresponds to the hydrophobic and hydrophilic domain, respectively [25]. As can be seen from Supplementary Figure S3, the hydrophilic domains in the composite membranes were uniformly distributed throughout. This stems out from the good interaction between the filler surface and the polymer backbone [18]. Thus, it can be stated that phase segregation existed at the nanoscale that might benefit the movement of the ions [25].

### 3.2. AEM Properties: IEC, WU, SR, GF, and Ionic Conductivity

The IEC represents the gravimetric charge density in the AEM. To explain the IEC difference between D-Cel composite and DG-Cel composite, the degree of quaternization (DQ) of quaternized cellulose (D-Cel and DG-Cel) was calculated from elemental analysis by using the equation as shown below (Supplementary Table S2) [60].

(7) $$\frac{{\mathrm{PC}}_{N}}{100}=\frac{\mathrm{DS}\times {\mathrm{MW}}_{N}\times {\mathrm{No}}_{N}}{{\mathrm{MW}}_{\mathrm{Cel}}+\mathrm{DS}\times {\mathrm{MW}}_{\mathrm{QR}}}$$

where ${\mathrm{PC}}_{N}$ is the weight percentage of nitrogen in the sample, DS is the degree of substitution, ${\mathrm{MW}}_{N}$ is the molecular weight of nitrogen, ${\mathrm{No}}_{N}$ is the number of nitrogen atoms in the quaternizing reagent, ${\mathrm{MW}}_{\mathrm{cel}}$ is the molecular weight of cellulose, and ${\mathrm{MW}}_{\mathrm{QR}}$ is the molecular weight of the quaternizing reagent. Based on the above equation the calculated value of DQ for DG-Cel and D-Cel were 0.08 and 0.38, respectively. The low DQ value of DG-Cel than D-Cel can be rooted to the lower substitution due to the steric hindrance of the bulky structure of the DG molecule [61]. The IEC of the composite showed a remarkable increase after the introduction of the D-Cel and DG-Cel (Table 1). The IEC of qPPO/D-Cel3 (1.46 mmol g^−1^) and qPPO/DG-Cel3 (0.87 mmol g^−1^) showed a 2 and 1.2 fold increase than pristine qPPO (0.71 mmol g^−1^), respectively. In all other cases, the IEC increased further with the increase in the quaternized cellulose contents as shown in Table 1. The qPPO/D-Cel7 showed the maximum IEC value of 1.66 mmol g^−1^, which is higher than qPPO/DG-Cel7 of 1.24 mmol g^−1^. This can be explained from the DQ value as mentioned above, as DG-Cel exhibited low DQ value, it imparts lower ionic content in the AEM at the same loading as that of D-Cel. This phenomenon is similar for different loadings of D-Cel and DG-Cel in the qPPO matrix (Table 1).

The WU of AEMs was also greatly influenced by the presence of the cellulose filler, which affected the ionic conductivity of the different composites. As shown in Table 1, the WU of pristine qPPO (17%) was increased to 41% and 56% with the incorporation of only 3 wt% of D-Cel and DG-Cel, respectively. As IEC of both qPPO/D-Cel and qPPO/DG-Cel increases, WU also increases, indicating that the WU is closely related to the IEC [62]. Unexpectedly, the WU of qPPO/DG-Cel (56–118%) was higher than that of qPPO/D-Cell (41–67%) in all cases for the same loadings of quaternized cellulose filler despite the lesser IEC value of qPPO/DG-Cel than qPPO/D-Cel as shown in Table 1. The reasons for this are probably due to the high basicity of the DG molecule [49] and the free space to store water resulting from a lower cross-linking degree of qPPO/DG-Cel than qPPO/D-Cel as evidenced by the gel fraction study [63] (Table 1). On the other hand, the WU of qPPO/D-Cel composites dropped after 7 wt% loading, resulting from the dense structure than the qPPO/DG-Cel and reduction of effective contact area between membrane and water because of the agglomeration of fillers at a high concentration [18,64].

The GF of qPPO was approximately 93%, which increased up to 99% and 95% after the incorporation of D-Cel and DG-Cel in the qPPO matrix, respectively, as shown in Table 1. The qPPO/D-Cel composites exhibited higher GF values than qPPO/DG-Cel composites, indicating that the D-Cel fillers were more efficiently cross-linked with the qPPO polymer matrix than DG-Cel fillers because of the higher DQ value of D-Cel than that for DG-Cel. Furthermore, the GF studies revealed that both D-Cel and DG-Cel were stable in the matrix without significant leaching. This is advantageous, as it has been reported that leaching of the fillers drastically affected the membrane performances and thus limited their practical applications [24]. It implies that qPPO/D-Cel composites might have more effectively enhanced the mechanical strength than qPPO/DG-Cel.

To further investigate the stability of the membranes under hydrated condition, the SR of the AEM samples was also determined. Since the more water that membrane holds, the more it swells, SR is strongly related to the WU. Excess swelling of the membrane should be restricted because it could lead to a weak mechanical strength or can deform itself during the fuel cell operations. Especially, qPPO/D-Cel7 that recorded the highest WU among the qPPO/D-Cel composites also recorded the highest SR at both inner-plane (17.59%) and through-plane (18.75%) compared to other qPPO/D-Cel composites (Table 1). In the case of qPPO/DG-Cel, 15 wt% loading of DG-Cel recorded the highest SR (SR_ip_: 21.94%, SR_tp_: 28.57%) among all other membranes with also the highest WU (118%). As the WU of both types of membranes increased, the SR also increased, and the SR of qPPO/DG-Cel (SR_ip_: 14–21%, SR_tp_: 16–28%) composites with greater WU was higher than that of qPPO/D-Cel (SR_ip_: 10–17%, SR_tp_: 6–18%) membranes (Table 1). Observation of less swelling of the qPPO/D-Cel membranes also supposed to arise from a more dense structure with a higher gel fraction compared to qPPO/DG-Cel membranes.

As depicted in Table 1, the ionic conductivity (σ) of pristine qPPO was 0.010 S cm^−1^, which increases considerably with the incorporation of D-Cel and DG-Cel fillers (Table 1). It can be seen that with only 3 wt% of D-Cel (i.e., qPPO/D-Cel3) the σ increased 2.9 folds compared to pristine qPPO. In the case of qPPO/DG-Cel3 (0.053 S cm^−1^), a 5.3-fold increase in σ was observed than qPPO only (0.010 S cm^−1^) at room temperature. As shown in Table 1, the ionic conductivity showed an improvement in trend with the increase in both D-Cel and DG-Cel loading. However, the ionic conductivity showed a decrease in trend after 7 wt% of quaternized cellulose loading. This decrease in ionic conductivity value indicates that the agglomeration of fillers at high contents loading hinders the movements of the hydroxyl ion by blocking the ionic channel [24]. In the case of qPPO/DG-Cel, the highest σ of 0.087 S cm^−1^, which is 8.7 and 1.5 times higher than pristine qPPO (0.010 S cm^−^^1^) and qPPO/D-Cel7 (0.058 S cm^−^^1^), was achieved at room temperature even with the lower IEC compared to qPPO/D-Cel composites. Three main reasons can be inferred: (1) high basicity of DG, better solvation by water [49], (2) less dense structure leads to higher WU, thus, improves the ionic conductivity because water is an ion transportation medium [16], (3) distinct phase separation of hydrophilic/hydrophobic domains that facilitates hydroxyl ion transport confirmed through the TEM and AFM images [65,66]. It was reported that the ion exchange membrane could enhance the ionic conductivity by establishing well-connected phase separation despite the low IEC [67]. Additionally, qPPO/D-Cel7 and qPPO/DG-Cel7 increased their ionic conductivity by 153% and 211%, respectively, compared to the pristine qPPO membrane after the cross-linking with cellulose filler (Supplementary Figure S4). When cross-linked with dibromo butane, the terminal amine groups are quaternized, increasing the charge density of the AEM, leading to enhanced ionic conductivity [51].

**Figure 4:**
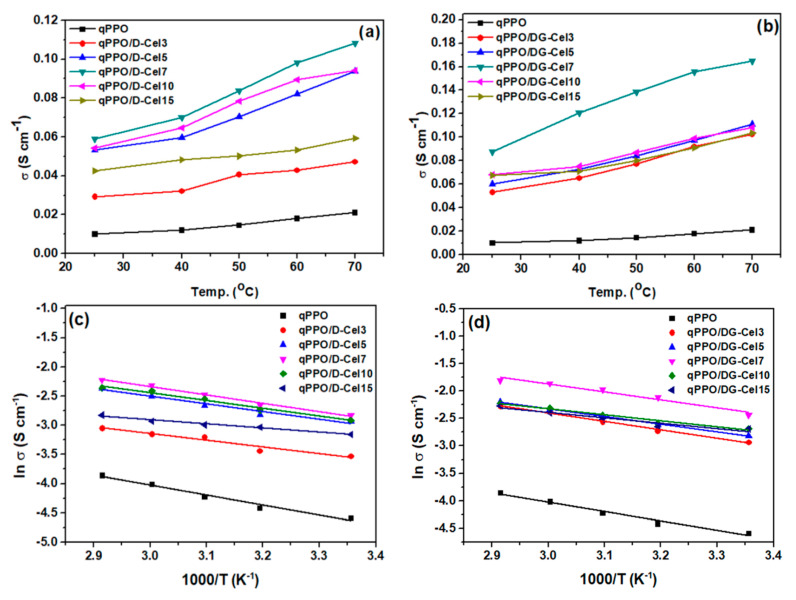
Ionic conductivity vs. temperature of (**a**) qPPO/D-Cel and (**b**) qPPO/DG-Cel membranes and Arrhenius plot of (**c**) qPPO/D-Cel and (**d**) qPPO/DG-Cel membranes.

The σ evaluated at different temperatures is presented in Figure 4a,b. Both qPPO/D-Cel and qPPO/DG-Cel composite membranes exhibited a pseudo linear relationship between σ and temperature in the range of 25–70 °C. The trend of ionic conductivity was similar in both qPPO/D-Cel composites and qPPO/DG-Cel composites. A 7 wt% loading of D-Cel and DG-Cel fillers achieved the highest ionic conductivity at all temperatures among any other qPPO/D-Cel composites and qPPO/DG-Cel composites, respectively (Figure 4a,b). The ionic conductivity, which depends on the temperature, also decreased with further loadings above 7 wt%, attributed to the blocking effect of the ionic channel as mentioned previously. The effect of temperature on σ has been explained by a theory that water content elevates with an increase in the temperature, which induces chain flexibility and ion diffusivity, thus, easing the migration of the hydroxyl ions [2]. In the scope of this study, the highest conductivity values for qPPO/D-Cel7 and qPPO/DG-Cel7 were 0.108 S cm^−1^ and 0.164 S cm^−1^, respectively, at 70 °C. The ionic conductivity in this work was found to be higher than most of the reported studies on similar types of anion exchange membranes (Supplementary Table S3). The activation energy (E_a_) was calculated from the slope of the Arrhenius plot (Figure 4c,d). Pristine qPPO membrane exhibited an E_a_ value of 14.1 kJ mol^−1^, which is much higher than that of the composite AEMs (6.69 to 11.73 kJ mol^−1^), indicating that a low barrier for hydroxide mobility was induced by the D-Cel and DG-Cel fillers.

### 3.3. Mechanical Property and Stabilities

**Figure 5:**
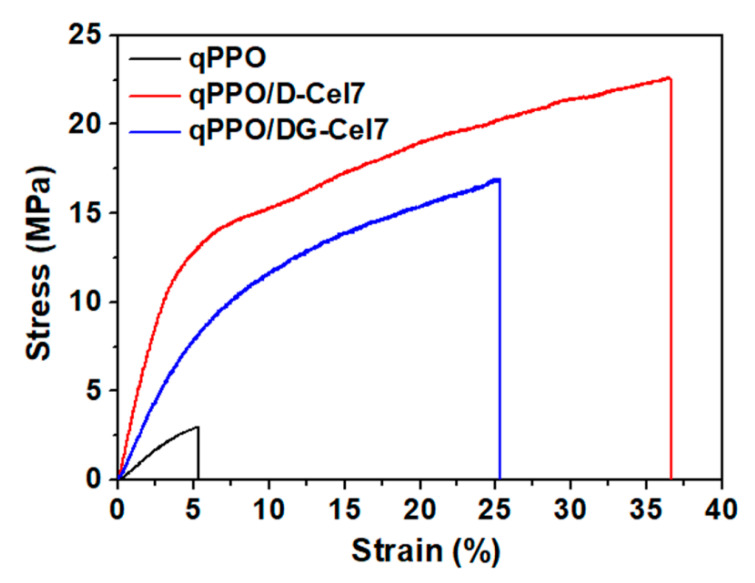
Tensile strength of membranes in a wet state.

The tensile properties of the composite membranes in the wet state were measured and compared as shown in the stress-strain curves (Figure 5). For comparison, the tensile strengths of qPPO and qPPO/D-Cel7 and qPPO/DG-Cel7 have also been presented. In the wet state, qPPO displayed a tensile strength of 3.03 MPa, whereas qPPO/D-Cel7 and qPPO/DG-Cel7 exhibited 22.68 and 16.96 MPa, respectively. A higher degree of cross-linking of qPPO/D-Cel than qPPO/DG-Cel membranes offers more resistance to chain slippage, thus requiring higher energy for breakage, as observed previously [4,68]. Moreover, the fillers with a high surface area enhance the interfacial interaction with the matrix, thereby limiting chain slippage [65]. Furthermore, the TGA measurements indicated that the composite membranes also showed better thermal stability than the pristine qPPO membrane. The weight loss occurred at 200–375 °C, which was attributed to the degradation of the cationic groups in the polymer chain, decreased by the presence of cellulose fillers as presented in Supplementary Figure S5.

**Figure 6:**
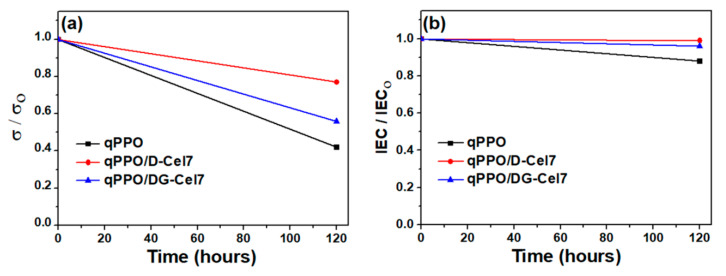
Alkaline stability of different anion exchange membranes (AEMs) in terms of the variations in (**a**) ionic conductivity and (**b**) ion exchange capacity (IEC) when treated with KOH solution (1 mol L^−1^) at 60 °C.

Figure 6 shows the alkaline stabilities of the membrane samples, which were estimated by observing the variations in their ionic conductivity and IEC in KOH (1 mol L^−1^) at 60 °C for 120 h. As shown in Figure 6a, the ionic conductivity of the pristine qPPO decreases by 58% after 120 h; on the other hand, qPPO/D-Cel7 and qPPO/DG-Cel7 decreases by 23% and 44%, respectively, of the initial value. The variations in IEC also showed a similar trend; qPPO, qPPO/D-Cel7, and qPPO/DG-Cel7 retained 88%, 99%, and 96% of the original IEC value after 120 h (Figure 6b). Thus, the composite membranes with quaternized cellulose are observed to maintain better stability than the pristine qPPO at 60 °C. Among the membranes, qPPO/D-Cel7 exhibited better alkaline stability, because of the electron pair sharing between the two nitrogen atoms inside the DABCO molecule [69]. Additionally, highly cross-linked membranes have been demonstrated to be much more stable under alkaline conditions [20,21]. Table 1 shows that the qPPO/D-Cel7 membrane has a lower WU and higher GF than qPPO/DG-Cel7, which can be considered less vulnerable to degradation because the former is denser and more cross-linked than the latter.

### 3.4. Fuel Cell Analysis

**Figure 7:**
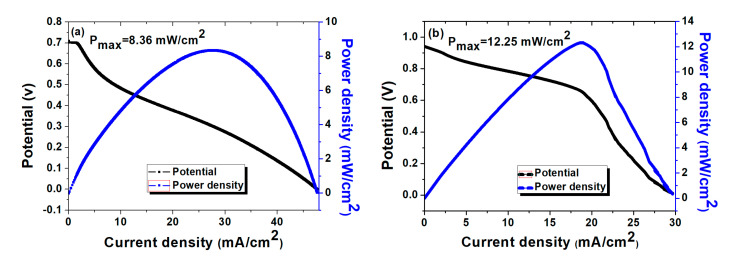
Polarization and power density curves of urea/O_2_ fuel cells operated using (**a**) qPPO/D-Cel7 and (**b**) qPPO/DG-Cel7 as AEM using urea (0.33 mol L^−1^) in aqueous KOH solution (3 mol L^−1^) as anolyte and moisturized O_2_ as the catholyte at 70 °C.

The applications of the synthesized composite AEMs in a urea/O_2_ fuel cell were evaluated using urea (0.33 mol L^−1^) in aqueous KOH solution (3 mol L^−1^) as anolyte and moisturized O_2_ as the catholyte. The experimental setup and fuel conditions were chosen based on previous reports from our laboratory [25]. A urea/O_2_ fuel cell with qPPO/D-Cel7 as an AEM showed an open-circuit voltage (OCV) of 0.71 V at 70 °C, and exhibited a maximum power density of 8.36 mW cm^-2^ at a current density of 27.44 mA cm^−2^ at 70 °C (Figure 7a). Meanwhile, the cell using qPPO/DG-Cel7 showed a higher OCV of 0.94 V than the qPPO/D-Cel7 due to reduced ohmic loss mainly caused by the high ionic conductivity [70,71], while a maximum power density of 12.25 mW cm^−2^ at a current density of 18.81 mA cm^−2^ was obtained at 70 °C (Figure 7b). The power density achieved in this study is observed to be higher than those reported by similar investigations on direct urea fuel cells (DUFCs) (Supplementary Table S4). Thus, the present study demonstrated that by using a suitable AEM, the performance of DUFCs can be enhanced under alkaline conditions. The current membranes can be further evaluated using a suitable high-performance catalytic system in the DUFCs.

## 4. Conclusions

This study evaluated the performance of composite AEMs made of PPO incorporated with cellulose as the filler. Cellulose was judiciously functionalized with two different amines viz., DABCO and DG. The ionic conductivity of the composite membrane showed that DG-functionalized cellulose exhibited higher value both at room temperature as well as at 70 °C. High ionic conductivities of 0.108 S cm^−1^ and 0.164 S cm^−1^ were achieved for qPPO/D-Cel7 and qPPO/DG-Cel7 membrane at 70 °C. The high basicity, great WU, and distinct ionic channels impart enhanced ionic conductivity to the qPPO/DG-Cel. Thus, this study demonstrated that by judiciously choosing the cationic group, the ionic conductivity of composite AEM can be enhanced by controlling the loading of the quaternized cellulose. The direct urea fuel cell measurements using both qPPO/D-Cel7 and qPPO/DG-Cel7 membrane exhibited peak power densities of 8.36 mW cm^−2^ and 12.25 mW cm^−2^ at 70 °C. The above result demonstrated the qPPO/D-Cel7 and qPPO/DG-Cel7 have the potential for practical applications.

## Figures and Tables

**Table 1:** Characteristics of the membranes.

Membrane	WU, %	$SR_{ip},\;\%$	$SR_{tp},\;\%$	IEC, mmol g^−1^	Gel Fraction, %	Ionic Conductivity (25 °C), S cm^−1^
qPPO	17	10.75	6.25	0.71	93	0.010
qPPO/D-Cel3	41	13.35	7.69	1.46	98.81	0.029
qPPO/D-Cel5	60	13.97	14.29	1.49	98.99	0.053
qPPO/D-Cel7	67	17.59	18.75	1.66	96.05	0.058
qPPO/D-Cel10	64	14.98	15.38	1.65	97.83	0.054
qPPO/D-Cel15	50	13.60	13.33	1.64	99.56	0.042
qPPO/DG-Cel3	56	14.84	16.33	0.87	94.2	0.053
qPPO/DG-Cel5	89	15.31	22.39	0.90	95.89	0.060
qPPO/DG-Cel7	91	18.52	22.89	1.24	95.65	0.087
qPPO/DG-Cel10	103	19.21	26.56	1.37	94.67	0.068
qPPO/DG-Cel15	118	21.94	28.57	1.45	94.36	0.067

## Supplementary Materials

The following are available online at https://www.mdpi.com/2073-4360/12/11/2676/s1, Scheme S1: Synthesis of guanidine and di-guanidine, Table S1. Composition in various membrane samples., Figure S1: ^1^H-NMR spectra of (a) DG, (b) Cel, (c) t-Cel, (d) D-Cel, (e) DG-Cel, (f) PPO, (g) bPPO, and (h) qPPO., Figure S2: SEM images of (a) Cel, (b) t-Cel, (c) D-Cel, and (d) DG-Cel., Figure S3: AFM image of (a,b) qPPO, (c,d) qPPO/D-Cel7, (e,f) qPPO/DG-Cel7., Table S2: Elemental analysis of D-Cel and DG-Cel, Figure S4: Effect of cross-linking on composite membranes., Table S3: Comparative data of AEMs, Figure S5: TGA graph of qPPO, qPPO/D-Cel7, and qPPO/DG-Cel7, Table S4: Comparative data of direct urea fuel cell.
