# Supplementary material for: A Composite Anion Conducting Membrane Based on Quaternized Cellulose and Poly(Phenylene Oxide) for Alkaline Fuel Cell Applications
Source: Polymers (Basel). 2020 Nov 12;12(11):2676. doi: 10.3390/polym12112676 (PMC7696857; doi:10.3390/polym12112676)
Supplement: Supplementary file 1 [file polymers-12-02676-s001.pdf]

Supporting information

A composite anion conducting membrane based on quaternized cellulose and poly(phenylene oxide) for alkaline fuel cell application

Dong Ho Kang, Gautam Das, Hyon Hee Yoon* and Il Tae Kim*

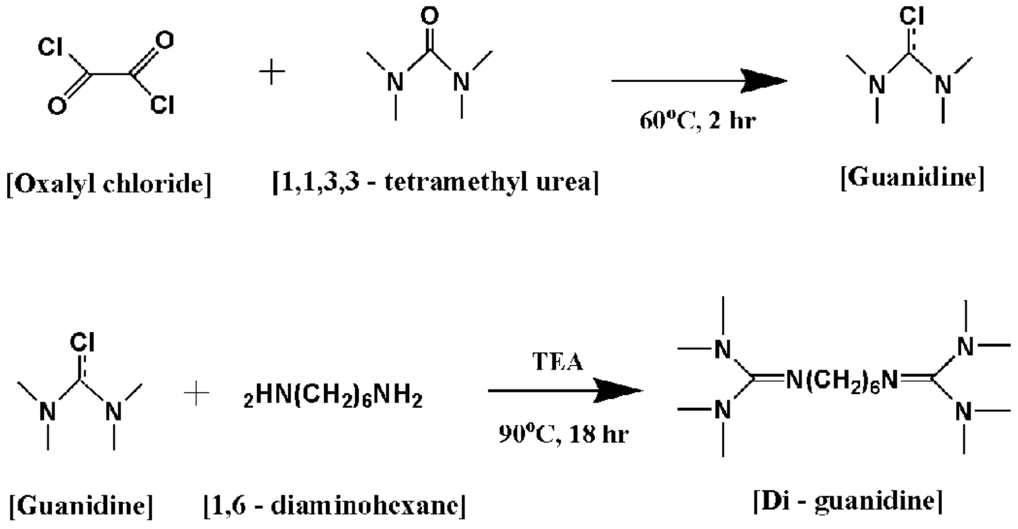

Scheme S1. Synthesis of guanidine and di-guanidine

Table S1. Composition in various membrane samples

Membrane	qPPO, g	D-Cel, g	DG-Cel, g
qPPO	1	-	-
qPPO/D-Cel3	1	0.03	-
qPPO/D-Cel5	1	0.05	-
qPPO/D-Cel7	1	0.07	-
qPPO/D-Cel10	1	0.10	-
qPPO/D-Cel15	1	0.15	-
qPPO/DG-Cel3	1	-	0.03
qPPO/DG-Cel5	1	-	0.05
qPPO/DG-Cel7	1	-	0.07
qPPO/DG-Cel10	1	-	0.10
qPPO/DG-Cel15	1	-	0.15

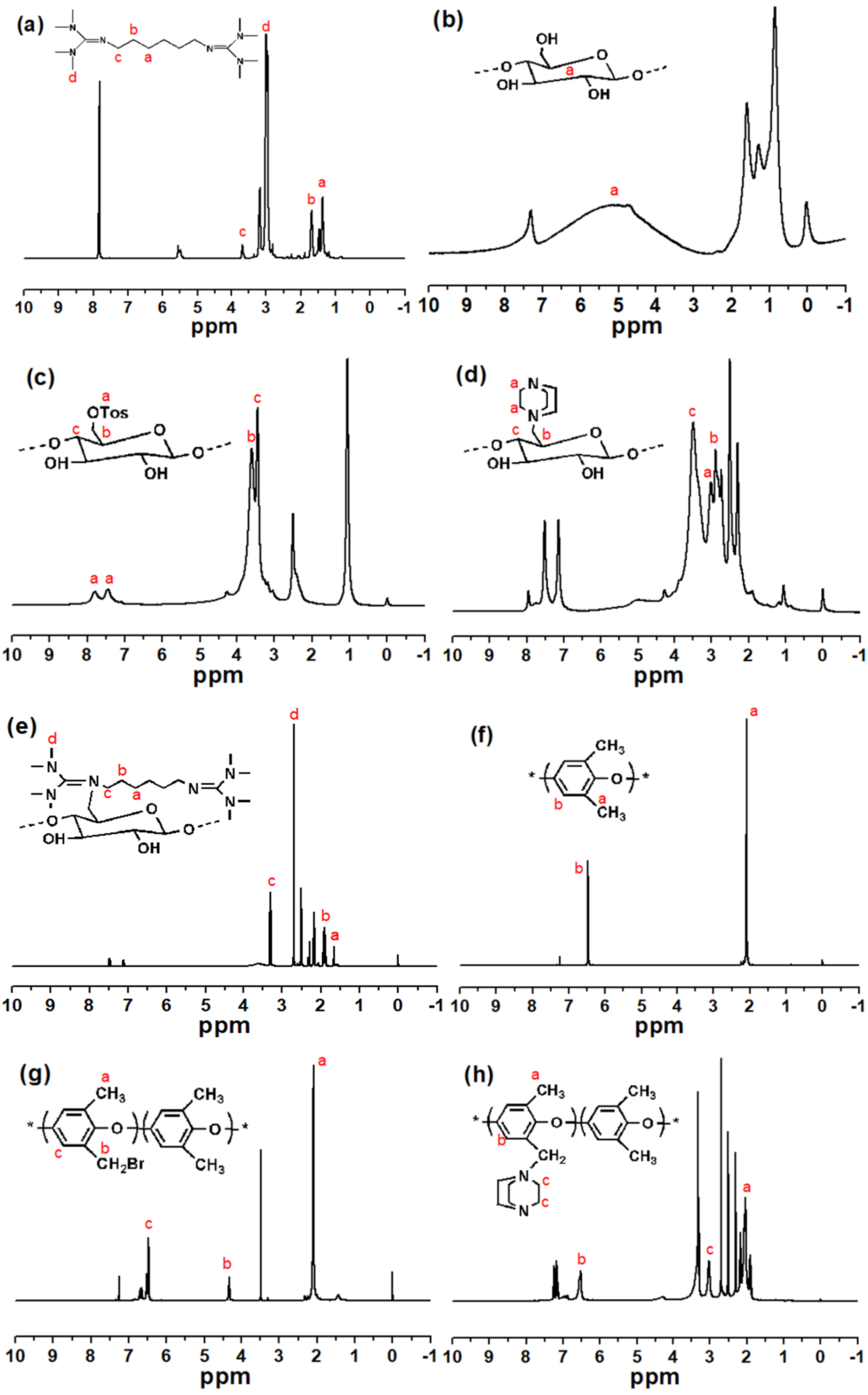

Figure S1. ^{1}H-NMR spectra of (a) DG, (b) Cel, (c) t-Cel, (d) D-Cel, (e) DG-Cel, (f) PPO, (g) bPPO, and (h) qPPO.

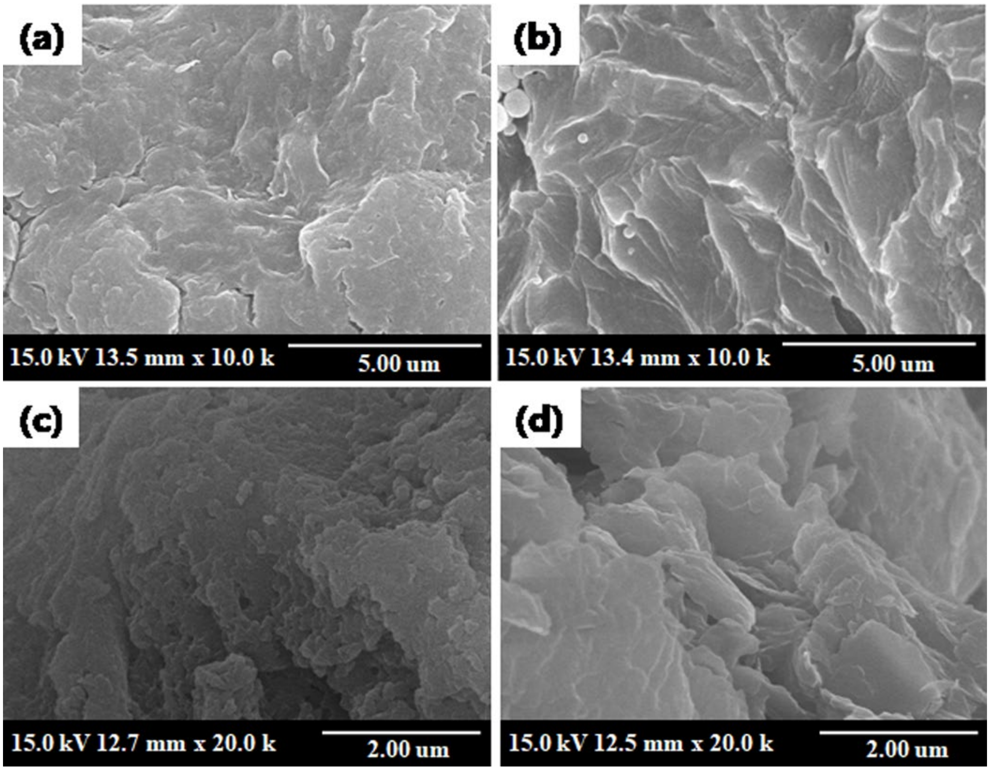

Figure S2. SEM images of (a) Cel, (b) t-Cel, (c) D-Cel, and (d) DG-Cel.

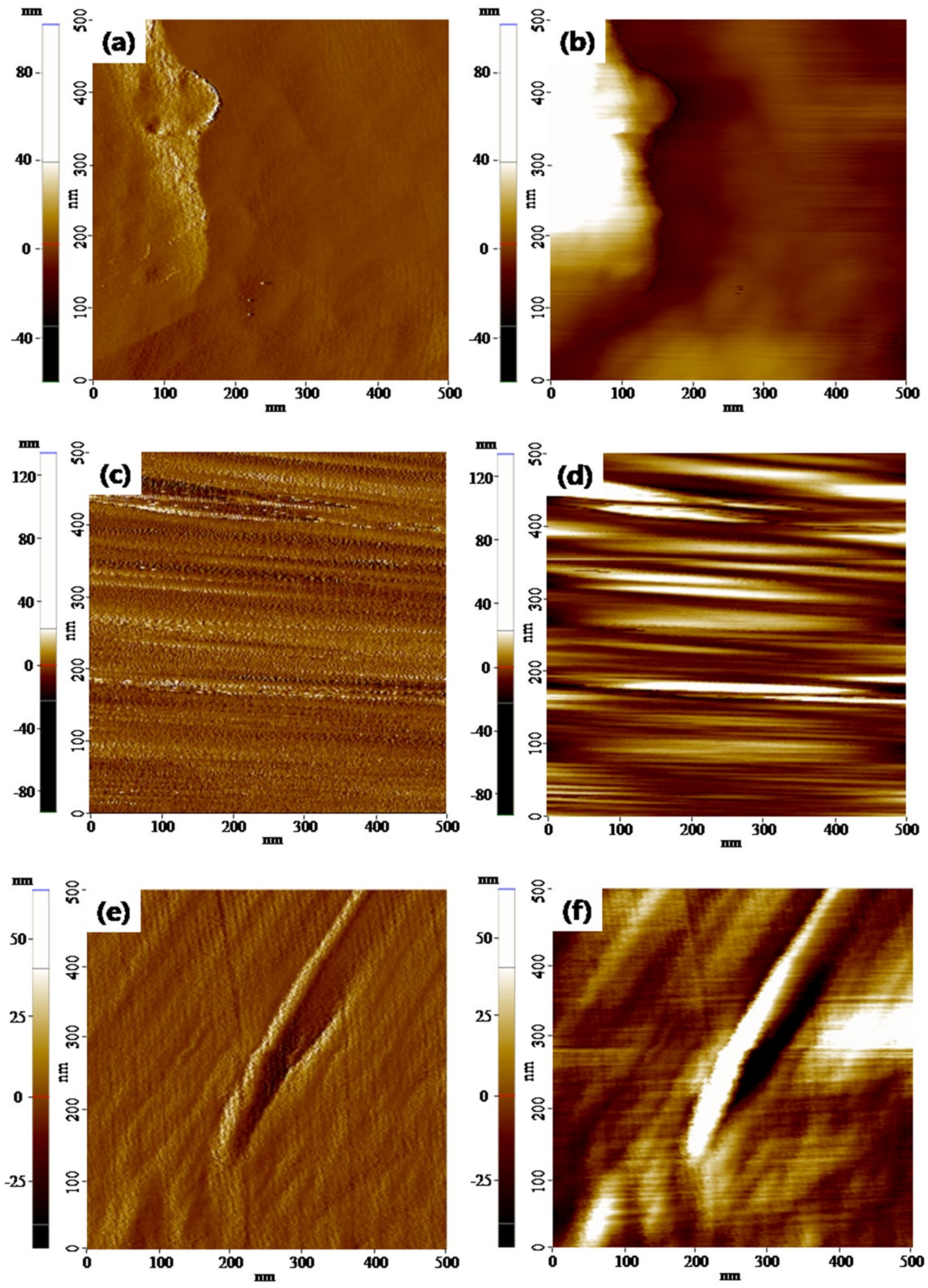

Figure S3. AFM image of (a, b) qPPO, (c, d) qPPO/D-Cel7, (e, f) qPPO/DG-Cel7.

Table S2. Elemental analysis of D-Cel and DG-Cel

Name	N (%)	C (%)	H (%)
D-Cel	5.19	47.91	6.825
DG-Cel	3.49	32.76	5.227

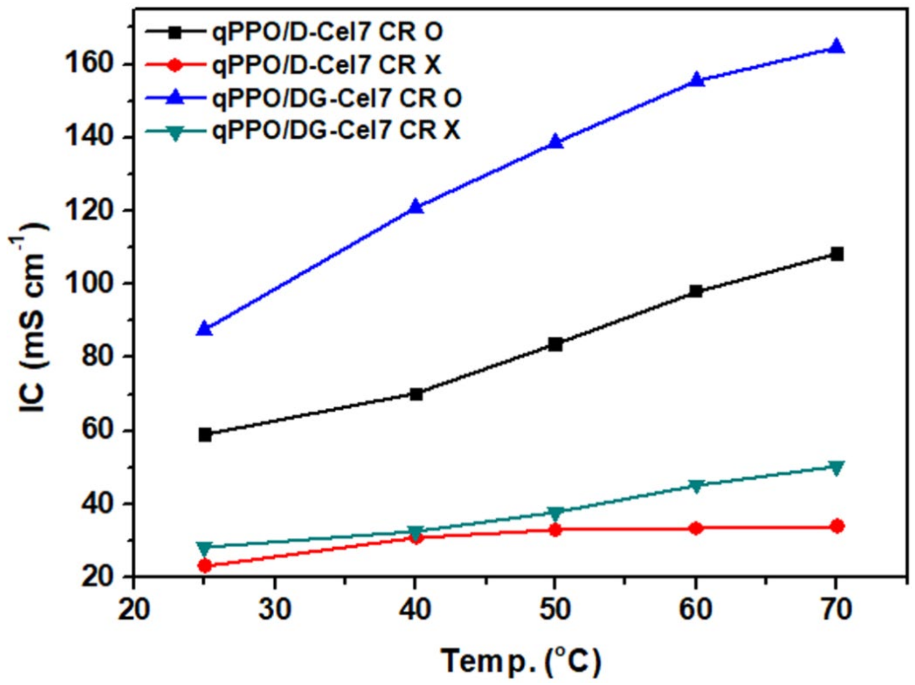

Figure S4. Effect of cross-linking on composite membranes.

Table S3. Comparative data of AEMs

Membrane	IC (RT), mS/cm	IC (>RT), mS/cm	IEC, mmol g^{-1}	WU, %	Ref.
PAES	~ 30	107 (80 °C)	2.2	50~60	[1]
Im-SiO_2/TA-PPO	~ 40	105 (80 °C)	3.15	145	[2]
PAES/Nano-ZrO_2	23.1	36.5 (80 °C)	1.82	28.1	[3]
CLQCPAES/nano-ZrO_2	16.2	55.2 (80 °C)	1.23	56.2	[4]
PSf/MMT	~ 15	47.3 (95 °C)	1.21	< 50	[5]
CS/QHNTs	5.56	17 (90 °C)	~0.4	79.2	[6]
QPSfDMC2	54	94 (70 °C)	2.34	124.72	[7]
QPPOQGO2	90.9	151 (80 °C)	2.25	74.28	[8]
QPSfQC15	74	128 (80 °C)	2.71	80.47	[9]
BG-BPS/PTFE	31	65 (60 °C)	1.14	29.4	[10]
Tri-QPESOH	45.9	130.9 (80 °C)	2.31	62	[11]
qPPO/D-Cel7	58.9	108.2 (70 °C)	1.66	67	This Work
qPPO/DG-Cel7	87.5	164.7 (70 °C)	1.24	91	This Work

* PAES = Poly(arylene ether sulfone), Im-SiO2/TA-PPO = Imidazolium-modified silica/Triple-ammonium side chain poly(phenylene oxide), CLQCPAES = Cross-linked multiblockcopoly(arylene ether sulfone), PSf = polysulfone, MMT = montmorillonite, CS = chitosan, QHNTs = Quaternized halloysite nanotubes, QPSfDMC = quaternized poly(sulfone) with N,N-dimethyl chitosan, QPPOQGO = quaternized poly(phenylene oxide) with modified graphene oxide, QPSfQC = DABCO polysulfone DABCO cellulose, BG-BPS = Bi-guanidinium bridged polysilsesquioxane, PTFE = poly tetra fluoro ethylene, QPESOH = quaternized poly (ether sulfone)

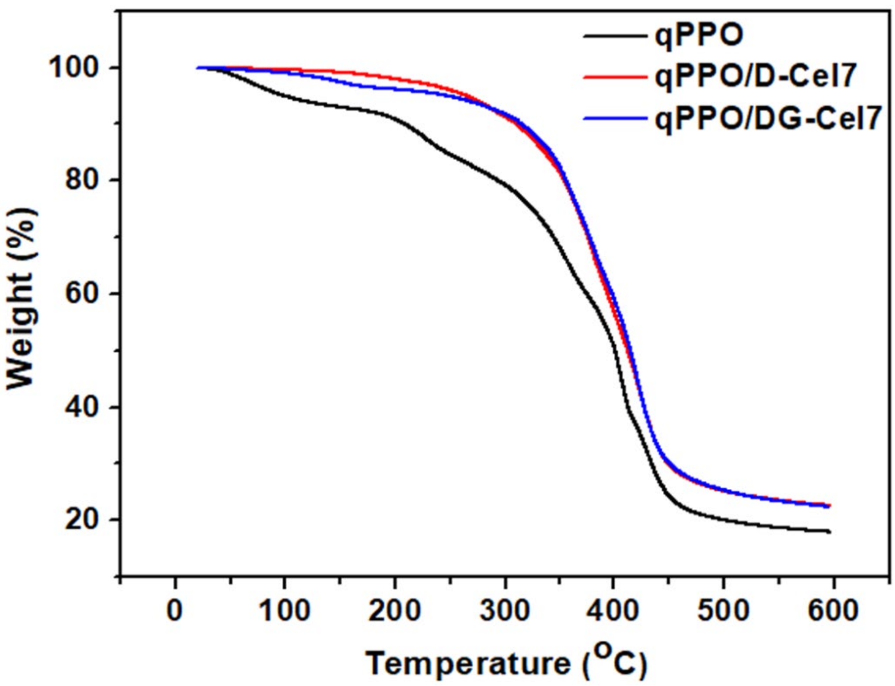

Figure S5. TGA graph of qPPO, qPPO/D-Cel7, and qPPO/DG-Cel7.

Table S4. Comparative data of direct urea fuel cell

Membrane	Fuel, Electrolyte	Catalyst (Anode-Cathode)	Power density (mW cm-2)	Ref
AMI-7001	0.33 mol L^{-1} Urea, 1 mol L^{-1} KOH	*Gr/Ni – Pt/C	4.06 x 10^{-3} (Room temperature)	[12]
Astom ACS A-5152	0.5 mol L^{-1} Urea	CuNi^PEDOT*PSS – Pt-B	1.88 (Room temperature)	[13]
FAA-3-50, Fumapem	0.33 mol L^{-1} Urea, 1 mol L^{-1} KOH	Pd-Ni/C – Pd/C	1.12 (Room temperature)	[14]
Fumasep FAA-3-50	0.33 mol L^{-1} Urea, 1 mol L^{-1} KOH	Ni/C – Mn_3O_4-Co_3O_4/MWCNT	0.4226 (50 °C)	[15]
QPSfDMC2	0.33 mol L^{-1} Urea, 1 mol L^{-1} KOH	Ni/C – PtRu/C	4.4 (70 °C)	[7]
QPPOQGO2	0.33 mol L^{-1} Urea, 1 mol L^{-1} KOH	Ni/C – PtRu/C	5.2 (60 °C)	[8]
qPPO/D-Cel7	0.33 mol L^{-1} Urea, 3 mol L^{-1} KOH	Ni/C – PtRu/C	8.36 (70 °C)	This work
qPPO/DG-Cel7	0.33 mol L^{-1} Urea, 3 mol L^{-1} KOH	Ni/C – PtRu/C	12.25 (70 °C)	This work

* Gr = graphene, MWCNT = multiwalled carbon nanotubes, AG = aerogel, PEDOT*PSS = poly(3,4-ethylenedioxythiophene) polystyrene sulfonate, Pt-B = Pt-Black

References

1. Lee, K.H.; Cho, D.H.; Kim, Y.M.; Moon, S.J.; Seong, J.G.; Shin, D.W.; Sohn, J.Y.; Kim, J.F.; Lee, Y.M. Highly conductive and durable poly(arylene ether sulfone) anion exchange membrane with end-group cross-linking. *Energy Environ. Sci.* **2017**, *10*, 275–285, doi:10.1039/c6ee03079c.
2. Chen, N.; Long, C.; Li, Y.; Wang, D.; Zhu, H. A hamburger-structure imidazolium-modified silica/polyphenyl ether composite membrane with enhancing comprehensive performance for anion exchange membrane applications. *Electrochim. Acta* **2018**, *268*, 295–303, doi:10.1016/j.electacta.2018.01.064.
3. Li, X.; Yu, Y.; Meng, Y. Novel quaternized poly(arylene ether sulfone)/nano-ZrO2 composite anion exchange membranes for alkaline fuel cells. *ACS Appl. Mater. Interfaces* **2013**, *5*, 1414–1422, doi:10.1021/am302844x.
4. Li, X.; Tao, J.; Nie, G.; Wang, L.; Li, L.; Liao, S. Cross-linked multiblock copoly(arylene ether sulfone) ionomer/nano-ZrO2 composite anion exchange membranes for alkaline fuel cells. *RSC Adv.* **2014**, *4*, 41398–41410, doi:10.1039/c4ra06519k.
5. Liao, X.; Ren, L.; Chen, D.; Liu, X.; Zhang, H. Nanocomposite membranes based on quaternized polysulfone and functionalized montmorillonite for anion-exchange membranes. *J. Power Sources* **2015**, *286*, 258–263, doi:10.1016/j.jpowsour.2015.03.182.
6. Shi, B.; Li, Y.; Zhang, H.; Wu, W.; Ding, R.; Dang, J.; Wang, J. Tuning the performance of anion exchange membranes by embedding multifunctional nanotubes into a polymer matrix. *J. Memb. Sci.* **2016**, *498*, 242–253, doi:10.1016/j.memsci.2015.10.005.
7. Das, G.; Kim, C.Y.; Kang, D.H.; Kim, B.H.; Yoon, H.H. Quaternized polysulfone cross-linked N,N-dimethyl chitosan-based anion-conducting membranes. *Polymers (Basel).* **2019**, *11*, 23–27, doi:10.3390/polym11030512.
8. Das, G.; Dongho, K.; Kim, C.Y.; Yoon, H.H. Graphene oxide crosslinked poly(phenylene oxide) nanocomposite as high-performance anion-conducting membrane. *J. Ind. Eng. Chem.* **2019**, *72*, 380–389, doi:10.1016/j.jiec.2018.12.040.
9. Das, G.; Park, B.J.; Yoon, H.H. A bionanocomposite based on 1,4-diazabicyclo-[2.2.2]-octane cellulose nanofiber cross-linked-quaternary polysulfone as an anion conducting membrane. *J. Mater. Chem. A* **2016**, *4*, 15554–15564, doi:10.1039/c6ta05611c.
10. Qu, C.; Zhang, H.; Zhang, F.; Liu, B. A high-performance anion exchange membrane based on bi-guanidinium bridged polysilsesquioxane for alkaline fuel cell application. *J. Mater. Chem.* **2012**, *22*, 8203–8207, doi:10.1039/c2jm16211c.
11. Chen, W.; Yan, X.; Wu, X.; Huang, S.; Luo, Y.; Gong, X.; He, G. Tri-quaternized poly (ether sulfone) anion exchange membranes with improved hydroxide conductivity. *J. Memb. Sci.* **2016**, *514*, 613–621, doi:10.1016/j.memsci.2016.05.004.
12. Yousef, A.; El-Newehy, M.H.; Al-Deyab, S.S.; Barakat, N.A.M. Facile synthesis of Ni-decorated multi-layers graphene sheets as effective anode for direct urea fuel cells. *Arab. J. Chem.* **2017**, *10*, 811–822, doi:10.1016/j.arabjc.2016.12.021.
13. Kaneto, K.; Nishikawa, M.; Uto, S.; Osawa, T. Direct urea fuel cells based on CuNi plated cloth as anode catalyst. *Chem. Lett.* **2018**, *47*, 1285–1287, doi:10.1246/cl.180566.
14. Yoon, J.; Lee, D.; Lee, Y.N.; Yoon, Y.S.; Kim, D.J. Solid solution palladium-nickel bimetallic anode catalysts by co-sputtering for direct urea fuel cells (DUFC). *J. Power Sources* **2019**, *431*, 259–264, doi:10.1016/j.jpowsour.2019.05.059.
15. Ngoc, T.; Pham, T.; Yoon, Y.S. Multiwalled Carbon Nanotubes for Cathode Catalyst in Urea Fuel Cell. **2020**, 1–13.
